# When the Nose Meets the Lab: Histopathological Analysis in Chronic Rhinosinusitis with Nasal Polyps for Routine Clinical Practice

**DOI:** 10.1007/s11882-024-01180-8

**Published:** 2024-10-07

**Authors:** Isam Alobid, Miguel Armengot-Carceller, Mayte Pinilla Urraca, Juan Maza-Solano, Isabel González Guijarro, Sebastián Umbria Jiménez, Pilar San Miguel Fraile, Joaquim Mullol

**Affiliations:** 1 Rhinology and Skull Base Unit, ENT Department, Hospital Clínic, Universitat de Barcelona, FRCB-IDIBAPS, CIBERES, Barcelona, Spain; 2 ENT Department, University and Polytechnic Hospital La Fe, University of Valencia, CIBERES, BMCG, IIS La Fe, Valencia, Spain; 3 Rhinology Unit, ENT Department, Puerta de Hierro University Hospital, University Autonoma Madrid, Majadahonda, Spain; 4 Rhinology Unit, ENT Department, University Hospital Virgen Macarena, Seville, Spain; 5 Department of Surgery, University of Seville, Seville, Spain; 6 ENT Department, Quironsalud Sagrado Corazón, Seville, Spain; 7 Rhinology Unit, ENT Department, Hospital Alvaro Cunqueiro, Vigo, Spain; 8 Pathology Department, Virgen Macarena University Hospital, Seville, Spain; 9 Head and Neck Pathology Unit, Pathology Department, Hospital Alvaro Cunqueiro, Vigo, Spain; 10 Rhinology Unit & Smell Clinic, ENT Department, Hospital Clinic Barcelona, FRCB-IDIBAPS, CIBERES, Barcelona, Spain

**Keywords:** Rhinosinusitis, Nasal Polyps, Eosinophils, Interleukin-5, Checklist, Professional Practice

## Abstract

**Purpose of review:**

We aimed to review the latest evidence regarding the value of tissue histopathological analysis in chronic rhinosinusitis with nasal polyps (CRSwNP) and to facilitate tissue analysis by proposing a pragmatic checklist for clinical settings.

**Recent findings:**

CRSwNP is a chronic inflammatory disease that severely impairs the patient’s quality of life. The severity of the disease can be correlated with nasal polyps enriched in eosinophils/IL-5 and, although ≥ 10 eosinophils per high power field are considered enough to determine an eosinophilic CRS, this cut-off value, the biopsy method, and the sampling location are still a matter of debate. Besides, tissue eosinophil values might also have some added value when combined with other cellular counts (e.g., eosinophil-to-lymphocyte ratio, Charcot-Leyden crystals). Structured histopathology analysis of sinonasal tissue—including, for instance, tissue remodelling biomarkers, fibrosis, and eosinophilic aggregates—has proven to be a valuable tool for healthcare professionals to identify different pheno-endotypes of CRSwNP and to improve the prioritisation of candidates to targeted therapies. Patients with CRSwNP are treated according to their severity with corticosteroids (intranasal and systemic), endoscopic sinus surgery, and/or biological therapy.

**Summary:**

A panel of expert ear, nose, and throat specialists and pathologists proposed a pragmatic checklist to improve the clinical practice around tissue analysis in CRSwNP, to facilitate communication between hospital-based healthcare professionals, and to standardize the evaluation of inflammatory biomarkers.

**Supplementary Information:**

The online version contains supplementary material available at 10.1007/s11882-024-01180-8.

## Introduction

Chronic rhinosinusitis (CRS) is a disease characterized by nose and paranasal sinuses inflammation with an estimated prevalence of 4% in the general population [1–3]. Nasal polyps are benign inflammatory masses arising from the middle meatus and paranasal sinuses. Clinical diagnosis of CRS with nasal polyps (CRSwNP) is made based on the presence of sinonasal symptoms (at least two) for more than 12 weeks of duration and with the visualization of nasal polyps in the nasal cavities by endoscopy [4, 5]. Cardinal symptoms include nasal congestion/blockage, anterior/posterior rhinorrhoea, pain or facial pressure, and impairment or loss of smell (hyposmia or anosmia) [1]. The ensuing quality of life of patients with CRSwNP is severely impaired, more than that of patients with congestive heart failure, angina, chronic obstructive pulmonary disease, and back pain [6]. In Europe, the total cost of CRSwNP is around €7,160 per patient per year, €1,501 due to direct costs (medical consultations and hospitalization) and €5,659 to indirect costs (mainly due to the loss of work productivity) [7]. The current treatment approach of CRSwNP revolves around three stages: i) appropriate medical treatment, ii) endoscopic sinus surgery (ESS) when no optimal response to medical treatment has been achieved, and iii) biological therapy in patients with severe uncontrolled disease not responding to standard care with medical and surgical treatment. The goal of treatment, supported by a growing consensus, is to maintain clinical control [8, 9]. Therefore, identifying the patients with poor control after a regular assessment is necessary to guide the therapeutic response [10].

The classical classification of CRS according to the presence or absence of nasal polyps has been modified by the European Position Paper on Rhinosinusitis and Nasal Polyps (EPOS) 2020 [5, 11]. Scientific evidence suggests that clustering CRS patients considering the inflammatory endotypes, such as the analysis of biomarkers, provides a clearer, more accurate description of the CRS subtypes than phenotype only [11]. Besides, recurrent CRSwNP patients needing revision ESS commonly present eosinophilic inflammation and predominant type-2 cytokines compared to nonrecurrent CRSwNP patients, highlighting once more the heterogeneity and complexity of the disease [12]. Consequently, structured histopathology has emerged as an interesting tool for ear, nose, and throat (ENT) specialists to identify the different pheno-endotypes of CRSwNP and enable them to better define targeted therapies accordingly [13].

Based on the currently available evidence, we aimed to provide some recommendations to undertake the histopathological analysis of samples from patients with nasal polyps to i) improve the clinical practice around the analysis of eosinophilic inflammation of nasal polyp tissue in CRSwNP, ii) streamline communication between specialists managing CRSwNP and pathologists, and iii) standardize the evaluation of inflammatory biomarkers from different centres. As further evidence emerges on the inflammatory endotypes of CRS, the proposed tissue analysis could eventually be expanded to all patients with chronic rhinosinusitis*,* not solely those with nasal polyps.

## Methods of Literature Search

A review of the literature was performed on July 2023, searching for articles published during the last 10 years in the MEDLINE database through PubMed with the following terms: "chronic rhinosinusitis" [Title/abstract] AND histopatholog* AND nasal polyp* AND eosinoph*. The search yielded 102 results that were reviewed at the title and abstract level. Those considered not relevant, mainly because they were out of the scope of the review, were excluded. To the 26 remaining publications, 13 were added by the authors based on their importance in the field, totalling, at the end, 39 publications analysed (Supplementary Figure S1).

Six ENT specialists and two pathologists worked in a face-to-face focus group session to develop the checklist. All participants could present their opinions, clinical practice examples and provided practical recommendations on how to perform histopathological analysis of CRSwNP tissue samples based on their current experience. As a result, they agreed on a clinical practice-oriented, pragmatic checklist with the most relevant inflammatory data from a histopathological analysis. They also proposed an extended version oriented to research settings based on a modified version of the seminal publication by Snidvongs et al*.* [14].

## Chronic Rhinosinusitis with Nasal Polyps: Current Histopathological Analysis

### Tissue Eosinophilia and Type-2 Inflammation on CRS Classification

CRSwNP is characterized by a type 2 inflammatory response [15], mainly regulated by IL-4, IL-5, and IL-13. Type 2 biomarkers include IgE, periostin, thymus and activation regulated chemokine, eotaxin-3, and eosinophilic cationic protein (ECP). The nasal mucosa of CRSwNP patients has a predominance of eosinophils, mast cells, Th-2 cells, ILC-2, and hyperplastic goblet cells, among others [16, 17]. The severity of the disease can be correlated with nasal polyps enriched in eosinophils/IL-5, predicting repeated surgeries in the patient’s lifetime [16]. EPOS has suggested classifying CRS as primary or secondary, further divided into localized or diffused based on anatomic distribution, and finally categorized by endotype dominance (Type 2 or non-Type 2 inflammation, local, mechanical). In primary, diffuse CRS, the clinical phenotypes are predominantly eosinophilic CRS (Type 2 inflammation) and non-eosinophilic CRS (non-Type 2), determined by the histologic quantification of the number of eosinophils [5]. Although the EPOS panel reached a consensus on ≥ 10 eosinophils per high power field (HPF) to determine an eosinophilic CRS [5], this cut-off value, the biopsy method, and the sampling location are still a matter of debate in the scientific literature [18, 19].

Eosinophilic inflammatory predominance has been identified as specific to CRSwNP, but the heterogeneity of CRS phenotypes has also been noted, with some CRSwNP patients presenting an inflammatory predominance other than eosinophilic. Recent studies suggest that the prevalence of non-eosinophilic CRSwNP is around 15% in Western countries [15, 20, 21]. Indeed, the presence of nasal polyps predicts high tissue eosinophilia, but this is not exclusively observed in CRSwNP patients; CRS without nasal polyps (CRSsNP) cases also present eosinophilic inflammation. There is some controversy on to what extent peripheral blood eosinophil levels could be a good surrogate marker of the presence of eosinophilia in the sinus mucosa, with tissue eosinophilia always pointed out as an unmet need in histopathology reports [14]. As aforementioned, the cut-off value to determine tissue eosinophilia (number of eosinophils per HPF) varies in the literature, ranging from 5 to 100 eosinophils per HPF, while some authors consider it should be potentially defined as the ratio of eosinophils to other inflammatory cells [19, 22].

Levels of tissue eosinophils and eosinophilic aggregates have been found to be different among CRSwNP patients with distinct clinical features (e.g., allergic fungal rhinosinusitis or NSAID-exacerbated respiratory disease [N-ERD]) [23]. In fact, computed tomography (CT) scores have revealed significant differences within CRSwNP, confirming the heterogeneity of this inflammatory condition. Accordingly, CT outcomes could be considered a diagnostic and prognostic tool, mainly to avoid generic treatments with poor outcomes [24]. Additionally, structural histopathology has been demonstrated to identify nasal polyps pheno-endotypes with a prevalent type of inflammatory cells (with different cell distribution patterns at the epithelium level) that could facilitate the recognition of adequate pharmacological treatments and follow-up approaches (e.g., biological drugs that inhibit specific cell populations) [25].

Tissue eosinophil values might also have some added value when combined with other cellular counts. For instance, the eosinophil-to-lymphocyte ratio in eosinophilic CRSwNP patients has been shown to be lower after ESS than before surgery, suggesting that nasal polyps could lead to eosinophilia. In addition, patients with eosinophilic CRSwNP have responded better to postoperative topical corticosteroid treatment, according to the reduction of their mean eosinophil-to-lymphocyte ratio and blood eosinophil count [26]. N-ERD and eosinophilic CRSwNP patients have been characterized histopathologically by the presence of high numbers of eosinophilic aggregates [13].

Charcot-Leyden crystals (CLC) are primary granules of the galectin superfamily and are released mainly by activated eosinophils. CLC levels in nasal brushings have been demonstrated to predict nasal polyp recurrence after surgery. Since tissue eosinophil counts has been identified as a good predictor of recurrence, and CLC protein concentrations have been positively correlated with eosinophil counts in nasal polyp tissue, CLC could be considered a good surrogate biomarker of eosinophilic inflammation [27, 28]. Likewise, periostin levels have also been shown to predict postoperative recurrence of nasal polyps. However, the periostin biomarker is not easily measured, which may hinder its wide applicability [29].

### Structured Histopathology and Tissue Biopsy

Nasal polyps have been subjected to histological evaluation and classified into oedematous (eosinophilic oedematous), intermediate (eosinophilic infiltrate, concomitant oedema, and collagen structure), and fibrous (predominant fibrous structure with variable cell infiltrates), although none of these subtypes was found to be predictive of a specific clinical status [30].

Tissue remodelling (basement membrane thickening, fibrosis, and squamous metaplasia) secondary to inflammation has been reported in the upper airways. Eosinophilic CRS and the presence of nasal polyps have been correlated with worse remodelling changes and mucosal damage. In fact, tissue eosinophilia and eosinophil activation have been more closely associated with clinical symptoms and have been shown to predict better mucosal damage than phenotypes based on the presence or absence of nasal polyps [31]. Clusters based on the combination of the 22-item sinonasal outcome test (SNOT-22) scores and tissue histopathology have been identified as more accurately classifying the distinct features of phenotypes than the clinical presence of nasal polyps [32]. Other potential biomarkers currently studied, beyond the number of eosinophils, include basement membrane thickening, subepithelial oedema, hyperplastic/papillary changes, fibrosis, eosinophilic aggregates, tissue remodelling markers, and fibroblasts, among others (Table 1) [33–38]. Interestingly, an interpretable prognosis network based on supervised deep learning, called ControlNet, which directly analysed standard haematoxylin and eosin-stained sections of polyp tissue, has been shown to effectively predict treatment outcomes of CRSwNP patients using tissue markers. Of note, this network highlighted the contributing role of eosinophil infiltration, goblet cell hyperplasia, glandular hyperplasia, squamous metaplasia, and fibrin deposition to poor outcomes in CRSwNP patients [39].

**Table 1:** Biomarkers/predictors of nasal polyps recurrence after endoscopic sinus surgery

Biomarker/predictor	Reference
Tissue eosinophil count	[27, 28, 40–43]
Neutrophilic and total inflammatory cell infiltration	[41]
Basement membrane thickness	[42]
Goblet cell hyperplasia	[42]
Charcot-Leyden crystals	[27, 28, 43]
Periostin levels	[29]
Degree of inflammation	[43]
Predominant type of inflammation	[43]
Hyperplastic seromucinous gland	[43]
Mucosal ulceration	[43]
Mucin containing eosinophil aggregates	[43]
Nasal polyps enriched in eosinophils/IL-5	[16]

IL-5: Interleukin-5

The histopathological assessment of tissue biopsy in CRS has several disadvantages, including its invasiveness and the lack of consensus on the definition of eosinophilia in CRS. Other biological markers have been considered, such as peripheral eosinophilia (limited by their broad variability, low specificity, and influenced by other comorbidities) or nasal cytology (a technique advised to assist in driving treatment selection) [44]. Also, ECP has been suggested as a surrogate marker of eosinophilia. ECP levels in uncinate tissue and nasal polyp homogenates have been demonstrated to correlate with radiographic severity (determined by CT score), but the correlation was stronger for uncinate tissue. The authors concluded that the degree of eosinophilia in uncinate tissue could be more representative of disease burden in CRSwNP at baseline and a better predictor of postoperative outcomes than that in nasal polyp homogenates [45]. This piece of research is a beautiful example that highlights how sample location can be a potential confounding factor when evaluating CRSwNP inflammation.

### Treatment Algorithms: The Impact of Histopathological Analysis on Treatment Choice

The Spanish consensus on the management of CRSwNP provides a treatment approach according to the severity of the disease (mild, moderate, or severe). Severity was defined based on the overall visual analogue scale score and/or the SNOT-22. The common basic treatment (Step 1) includes intranasal corticosteroids and saline nasal rinsing. For moderate CRSwNP cases showing a lack of control for three to six months, a short course of oral or systemic corticosteroids is recommended, with a maximum of two courses per year (Step 2). For severe patients showing a lack of control during three to six months, ESS (Step 3) is proposed. After primary surgery, if the patient shows severe, uncontrolled disease, initiation with biological therapy or revision surgery could be considered (Step 4) [10]. Severe uncontrolled patients who are candidates for biologics are evaluated along three dimensions: impact of CRSwNP symptoms, presence of lower airway disease, and level of inflammatory biomarkers (among which the detection of local inflammation markers is recommended). Patients displaying ≥ 10 eosinophils per HPF are considered candidates for biologic therapy.

## Structured Histopathology Checklist for Reporting CRSwNP Findings

Structured histopathology analysis has emerged as a valuable tool in the field of CRS in the context of clinical research [14]. Whereas some histopathological features are consistently evaluated in articles reporting structured histopathology analysis (overall degree of inflammation, eosinophil count, sub-epithelial oedema, squamous metaplasia, fibrosis, and eosinophil aggregates), other parameters are not always included (basement membrane thickening, fungal elements, goblet cell hyperplasia, inflammatory predominance, CLC, neutrophilic infiltrate, fibroblasts, eosinophil extracellular traps, hyperplastic/papillary change, and mucosal ulceration) [14, 37, 38, 46–50].

Considering time and resource constraints, a pragmatic, clinical practice-oriented checklist for histopathological analysis in CRSwNP (Table 2 and supplementary material) was proposed, along with an extended version suggesting the evaluation of other morphological features that could bring additional information useful either for clinical or research purposes (Table 3 and supplementary material). A detailed protocol on how to perform the absolute eosinophil count in CRSwNP patients and a downloadable, ready-to-use version of both checklists is presented in the Supplementary Material.

**Table 2:** Routine clinical practice pragmatic checklist for CRSwNP histopathological analysis

Item	Description
Inflammation level^a^	Low / moderate / severe
Eosinophil count per high-power field (HPF)^b^	If the value is:
	< 10 cells/HPF: report exact number
	≥ 10 – 50 cells/HPF: report exact number
	> 50 – 100 cells/HPF: report number rounded to the nearest tens
	> 100 cells/HPF: report > 100 cells/HPF
Inflammatory predominance	Indicate the inflammatory predominant cell type by percentage (eosinophils, lymphocytes, plasmatic cells, and neutrophils)

^a^ Based on the extent of overall cellular inflammatory infiltration observed in tissue. ^b^We recommend reporting the result as the mean value of the analysis of three different locations within the tissue

**Table 3:** Extended checklist for CRSwNP histopathological analysis based on previous literature [14, 47–49] and expert consensus

Tissue sample		
	**Specimen (select all that apply)**	□ Nasal cavity
		□ Septum
		□ Floor
		□ Lateral wall
		□ Vestibule
		□ Paranasal sinus(es), maxillary
		□ Paranasal sinus(es), ethmoid
		□ Paranasal sinus(es), frontal
		□ Paranasal sinus(es), sphenoid
		□ Respiratory mucosa
		□ Mucoserous glands
		□ Bone
		□ Others specify:
		□ Not specified
	**Received:**	□ Fresh
		□ In formalin
		□ Others (specify):
Tissue analysis		
	**Inflammation level**	□ Low
		□ Moderate
		□ Severe
	**Eosinophil count per high-power field (HPF)**	□ < 10 cells/HPF: report exact number
		□ ≥ 10 – 50 cells/HPF: report exact number
		□ > 50 – 100 cells/HPF: report number rounded to the nearest tens
		□ > 100 cells/HPF
	**Inflammatory predominance**	Indicate the inflammatory predominant cell type by percentage:
		□ Eosinophils:
		□ Lymphocytes:
		□ Plasmatic:
		□ Neutrophils:
	**Charcot-Leyden crystals**	□ Absent
		□ Present
	**Fibrosis**	□ Absent
		□ Partial
		□ Extensive
	**Basement membrane thickening**	□ Absent
		□ < 7.5 µM
		□ 7.5 – 15 µM
		□ > 15 µM
	**Eosinophil aggregates**	□ Absent
		□ Present
	**Goblet cell metaplasia**	Identifiable in a × 400 field:
		□ < 3 cells
		□ 3 – 10 cells
		□ 11 – 20 cells
		□ > 20 cells
	**Neutrophilic infiltrate**	□ Absent
		□ Focal
		□ Diffuse
	**Sub-epithelial oedema**	□ Absent
		□ Mild (focal or perivascular only)
		□ Moderate (distortion of mucosal structure)
		□ Severe (diffuse/polypoid change)
	**Formation of submucosal glands**	□ Absent
		□ Present
	**Hyperplastic/papillary change**	□ Absent
		□ Present
	**Eosinophil degranulation or cytolysis**	□ Absent
		□ Mild
		□ Moderate
		□ Severe (signs of extracellular trap cell death [Eosinophil ETosis])
	**Fungal elements**	□ Absent
		□ Present

In routine clinical practice, the biopsy aims to establish or confirm the inflammatory profile of the disease. A suitable candidate for a biopsy procedure would be a severe patient with non-controlled CRS and recurrence of polyps after a primary surgery. The information yielded from the pragmatic checklist could assist the ENT and other specialists in the suitability of initiating a biological treatment. Inflammation levels, tissue eosinophil counts, and the inflammatory predominance in the tissue would offer relevant information on the appropriateness of the proposed treatment and inform about the patient’s prognosis.

The exact location in the nasal cavity where the sample should be taken was a topic of debate among the panel. This is a relevant question, considering that nasal polyps may not be present after initiating biological treatment or in the following months after a surgical intervention. It was suggested that sample collection must be taken systematically from the same nasal location to allow for evaluation of patient histopathological changes over time. Polyp tissue, or in its absence, tissue from the nasal location where polyps are originated (mucosa of the middle meatus, middle turbinate, or uncinate process) were defined as the optimal location for sample collection [51]. Accessibility of uncinate tissue might be challenging for an in-office sample collection procedure. If uncinate tissue is unavailable because of a previous resection, the tissue sample could be collected right at the start of the middle turbinate (albeit also challenging for an in-office procedure).

## Conclusions

CRSwNP is a heterogeneous and complex disease characterized by an eosinophilic inflammatory predominance. Structured histopathological analysis of tissue has proven to be a reliable tool for classifying pheno-endotypes and identifying patients for targeted therapies. Here, a panel of expert ENT specialists and pathologists proposed a pragmatic checklist for the histopathological analysis of CRSwNP patients in routine clinical practice, as well as an extended version of the checklist for research purposes. We expect these checklists will help fulfil the goals of improving the analysis of CRSwNP tissue, facilitating communication between healthcare professionals and standardizing the evaluation of inflammatory biomarkers.

## Supplementary Information

Below is the link to the electronic supplementary material.

Supplementary file1 (PDF 237 KB)

Supplementary file2 (JPG 29 KB)

Supplementary file3 (DOCX 1596 KB)

## Data Availability

No datasets were generated or analysed during the current study.
