# Supplementary material for: When the Nose Meets the Lab: Histopathological Analysis in Chronic Rhinosinusitis with Nasal Polyps for Routine Clinical Practice
Source: Curr Allergy Asthma Rep. 2024 Oct 7;24(12):657–65. doi: 10.1007/s11882-024-01180-8 (PMC11485015; doi:10.1007/s11882-024-01180-8)
Supplement: Supplementary file 1 — Supplementary file1 (PDF 237 KB) [file 11882_2024_1180_MOESM1_ESM.pdf]

CLINICAL PRACTICE PRAGMATIC CHECKLIST for Chronic Rhinosinusitis with Nasal Polyps

HISTOPATHOLOGICAL ANALYSIS

DATE

PATIENT DATA ....

TO BE COMPLETED BY THE HEALTHCARE SPECIALIST

PATIENT INFORMATION

AGE .... years

AGE OF ONSET OF CRSwNP .... years

CRSwNP Severity
- ☐ Low
- ☐ Moderate
- ☐ Severe

Does the patient have a history of nasal surgery?

☐ No ☐ Yes if yes, number of interventions ....

Current treatment

Other relevant information

TISSUE SAMPLE

Specimen (select all that apply):

- ☐ **Nasal cavity**
  - ☐ Septum ☐ Floor ☐ Lateral wall ☐ Vestibule
- ☐ **Paranasal sinus(es)**
  - ☐ Maxillary ☐ Ethmoid ☐ Frontal ☐ Sphenoid
- ☐ **Respiratory mucosa** ☐ **Mucoserous glands** ☐ **Bone**
- ☐ **Not specified** ☐ **Others specify:** ....

TO BE COMPLETED BY THE PATHOLOGIST

Received: ☐ Fresh ☐ In formalin ☐ Others (specify): ....

TISSUE ANALYSIS

Inflammation level: ☐ Low ☐ Moderate ☐ Severe

Eosinophil count per high-power field:
- ☐ < 10 cells/HPF (report exact number):..........................
- ☐ ≥ 10 - 50 cells/HPF (report exact number): ....
- ☐ > 50 – 100 cells/HPF (report number rounded to the nearest tens): ....
- ☐ > 100 cells/HPF

Inflammatory predominance (indicate predominant cell type by %)
- ☐ Eosinophils: ....
- ☐ Lymphocytes: ....
- ☐ Plasmatic cells: ....
- ☐ Neutrophils: ....

COMMENTS

EXTENDED CHECKLIST for Chronic Rhinosinusitis with Nasal Polyps

HISTOPATHOLOGICAL ANALYSIS

DATE

PATIENT DATA ....

TO BE COMPLETED BY THE HEALTHCARE SPECIALIST

PATIENT INFORMATION

AGE .... years

AGE OF ONSET OF CRSwNP .... years

CRSwNP Severity
- ☐ Low
- ☐ Moderate
- ☐ Severe

Does the patient have a history of nasal surgery?

☐ No ☐ Yes if yes, number of interventions ....

Current treatment

Other relevant information

TISSUE SAMPLE

Specimen (select all that apply):

☐ **Nasal cavity**	☐ Septum	☐ Floor	☐ Lateral wall	☐ Vestibule
☐ **Paranasal sinus(es)**	☐ Maxillary	☐ Ethmoid	☐ Frontal	☐ Sphenoid

☐ **Respiratory mucosa** ☐ **Mucoserous glands** ☐ **Bone**

☐ **Not specified** ☐ **Others specify:** ....

TO BE COMPLETED BY THE PATHOLOGIST

Received: ☐ Fresh ☐ In formalin ☐ Others (specify): ....

TISSUE ANALYSIS

Inflammation level: ☐ Low ☐ Moderate ☐ Severe

Eosinophil count per high-power field:
- ☐ < 10 cells/HPF (report exact number):..
- ☐ ≥ 10 - 50 cells/HPF (report exact number): ....
- ☐ > 50 – 100 cells/HPF (report number rounded to the nearest tens): ....
- ☐ > 100 cells/HPF

Inflammatory predominance
(indicate predominant cell type by %)
- ☐ Eosinophils: ....
- ☐ Lymphocytes: ....
- ☐ Plasmatic cells: ....
- ☐ Neutrophils: ....

**Basement membrane thickening**	☐ Absent	☐ < 7,5 μM	☐ 7,5 -15 μM	☐ > 15 μM
**Goblet cell metaplasia** Identifiable in a x400 field:	☐ < 3 cells	☐ 3-10 cells	☐ 11-20 cells	☐ > 20 cells
**Sub-epithelial edema**	☐ Absent	☐ Mild[a]	☐ Moderate[b]	☐ Severe[c]
**Eosinophil degranulation or cytolysis**	☐ Absent	☐ Mild	☐ Moderate	☐ Severe[d]

a Mild (focal or perivascular only); **b** Moderate (distortion of mucosal structure); **c** Severe (diffuse/polypoid change); **d** Severe (signs of extracellular trap cell death (eosinophil ETosis)

Fibrosis	☐ Partial	☐ Extensive	☐ Absent
Neutrophilic infiltrate	☐ Focal	☐ Diffuse	☐ Absent

	PRESENT	ABSENT
Charcot-Leyden crsytals	☐	☐
Eosinophil aggregates	☐	☐
Formation of submucosal glands	☐	☐
Hyperplastic/papillary change	☐	☐
Fungal elements	☐	☐

COMMENTS
