# Supplementary figures and images for: When the Nose Meets the Lab: Histopathological Analysis in Chronic Rhinosinusitis with Nasal Polyps for Routine Clinical Practice
Source: Curr Allergy Asthma Rep. 2024 Oct 7;24(12):657–65. doi: 10.1007/s11882-024-01180-8 (PMC11485015; doi:10.1007/s11882-024-01180-8)

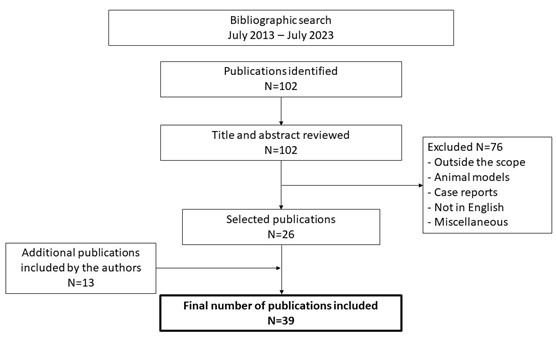

Supplement: Supplementary file 2 — Supplementary file2 (JPG 29 KB) [file 11882_2024_1180_MOESM2_ESM.jpg]
