# Supplementary material for: When the Nose Meets the Lab: Histopathological Analysis in Chronic Rhinosinusitis with Nasal Polyps for Routine Clinical Practice
Source: Curr Allergy Asthma Rep. 2024 Oct 7;24(12):657–65. doi: 10.1007/s11882-024-01180-8 (PMC11485015; doi:10.1007/s11882-024-01180-8)

***When the nose meets the lab:*** ***Histopathological analysis in chronic rhinosinusitis with nasal polyps for routine clinical practice***

**Supplementary Material**

| **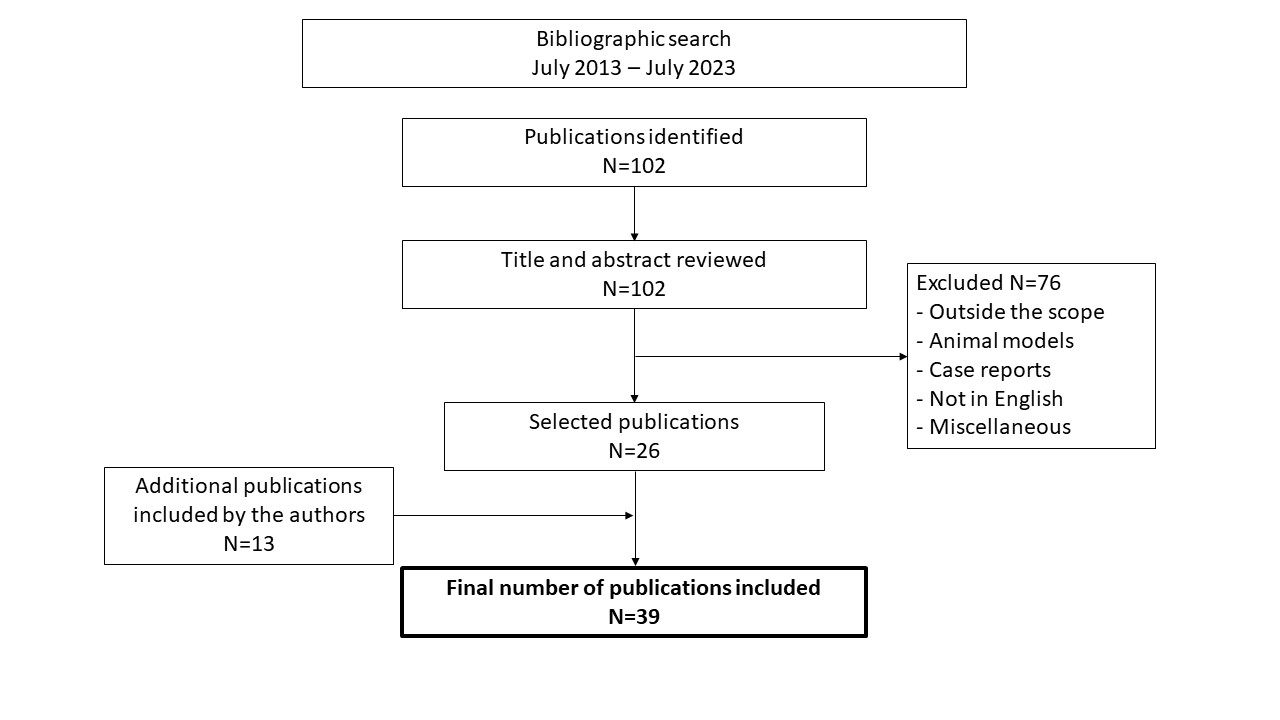** |
| --- |
| **Supplementary Figure S1.** Flowchart of literature selection for the review. |

| **Protocol for absolute eosinophil count in chronic rhinosinusitis with nasal polyps (CRSwNP) patient tissue** [1] |
| --- |
| Tissue eosinophilia in CRSwNP is usually measured using samples taken either during an endoscopic sinus surgery or in-office using local anaesthesia.  Tissue specimens are fixed in 10% buffered formalin, processed, embedded in paraffin, cut into 4 µm and stained with haematoxylin-eosin. Eosinophil count in tissue is carried out at high power field (x400). Cell count should be performed at three hot spots (areas where eosinophils are concentrated). Reported values are recommended to be the result of the mean value of the analysis of these three different locations. |

1 Tokunaga T, Sakashita M, Haruna T, et al (2015) Novel scoring system and algorithm for classifying chronic rhinosinusitis: the JESREC Study. Allergy 70(8):995–1003. https://doi.org/10.1111/all.12644

**Clinical practice, pragmatic checklist for CRSwNP histopathological analysis (downloadable version)**


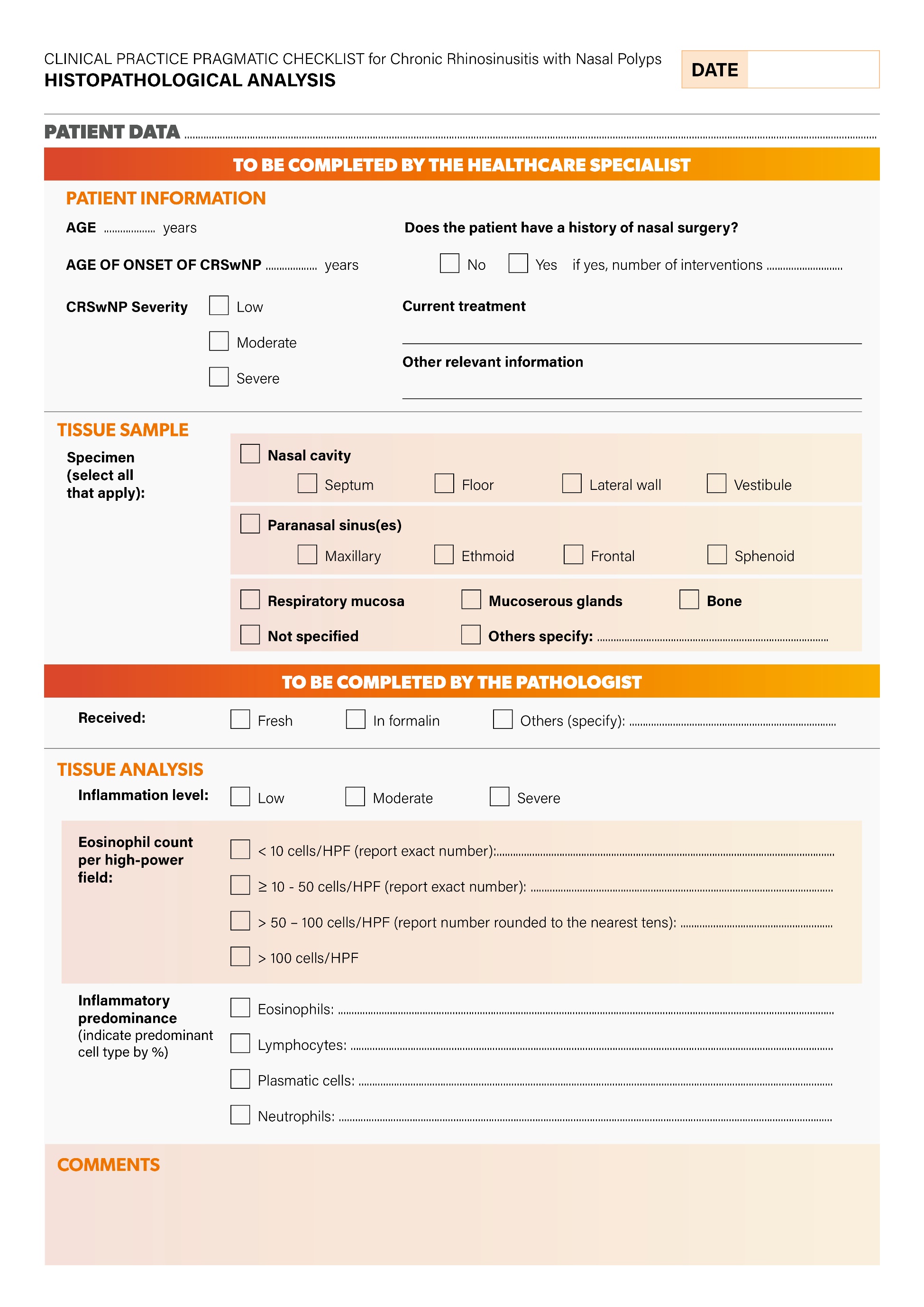


**Extended checklist for CRSwNP histophatological analysis
(downloadable version)**


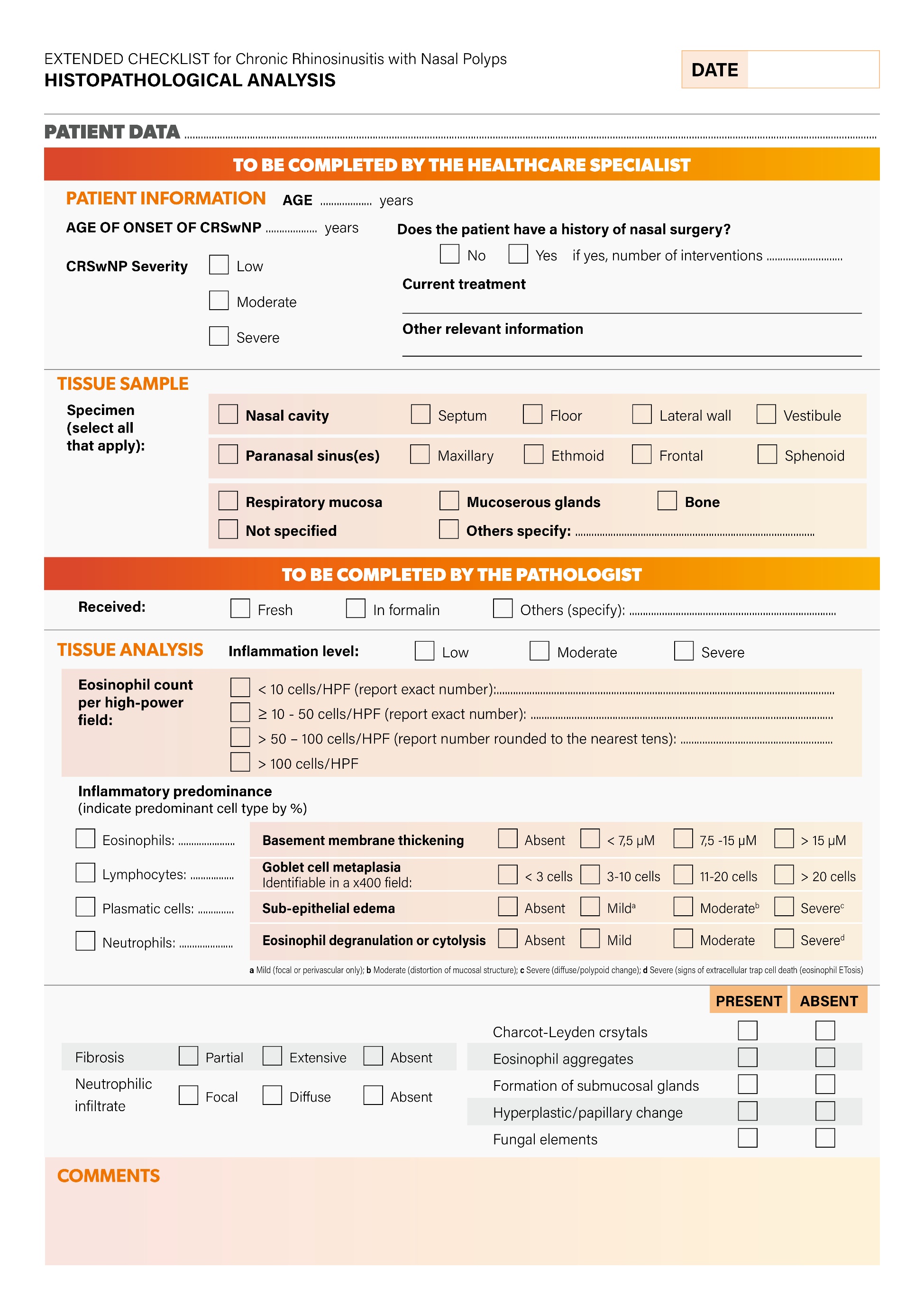

Supplement: Supplementary file 3 — Supplementary file3 (DOCX 1596 KB) [file 11882_2024_1180_MOESM3_ESM.docx]
